# Unravelling Glucoraphanin and Glucoerucin Metabolism across Broccoli Sprout Development: Insights from Metabolite and Transcriptome Analysis

**DOI:** 10.3390/plants13060750

**Published:** 2024-03-07

**Authors:** Jiansheng Wang, Yusen Shen, Xiaoguang Sheng, Huifang Yu, Mengfei Song, Qiaomei Wang, Honghui Gu

**Affiliations:** 1 Institute of Vegetables, Zhejiang Academy of Agricultural Sciences, Hangzhou 310021, China; wangjs@zaas.ac.cn (J.W.); shenyusen@zaas.ac.cn (Y.S.); xguang@zaas.ac.cn (X.S.); yuhf@zaas.ac.cn (H.Y.); smf993@163.com (M.S.); 2 Key Laboratory of Horticultural Plant Growth, Development and Quality Improvement, Department of Horticulture, Zhejiang University, Hangzhou 310058, China; qmwang@zju.edu.cn

**Keywords:** broccoli sprout, glucosinolate, RNA-seq, sulforaphane, erucin

## Abstract

Variations in the concentration of glucoraphanin (GRA) and glucoerucin (GER), as well as the corresponding breakdown products, isothiocyanates (ITCs) and nitriles, were investigated during the growth of broccoli sprouts. The concentrations of GRA and GER decreased sharply from 33.66 µmol/g to 11.48 µmol/g and 12.98 µmol/g to 8.23 µmol/g, respectively, after seed germination. From the third to the seventh day, both GRA and GER were maintained as relatively stable. The highest concentrations of sulforaphane (17.16 µmol/g) and erucin (12.26 µmol/g) were observed on the first day. Hereafter, the concentrations of nitrile hydrolyzed from GRA or GER were higher than those of the corresponding ITCs. Moreover, the ratio of sulforaphane to sulforaphane nitrile decreased from 1.35 to 0.164 from 1 d to 5 d, with a similar trend exhibited for erucin/erucin nitrile after 2 d. RNA-seq analysis showed that *BolMYB28* and *BolCYP83A1*, involved in aliphatic glucosinolate (GSL) biosynthesis, remained largely unexpressed until the third day. In contrast, the genes operating within the GSL-myrosinase hydrolysis pathway were highly expressed right from the beginning, with their expression levels increasing significantly after the third day. Additionally, we identified two *BolESPs* and six *BolNSPs* that might play important roles in promoting the production of nitriles during the development of broccoli sprouts.

## 1. Introduction

Broccoli sprouts are more and more popular for their high nutritional value, primarily due to their abundant glucosinolates (GSLs) and the resulting isothiocyanates (ITCs) upon breakdown [1]. GSLs are thioglucosides widely present in *Brassica oleracea* vegetables and wild species [2]. According to their amino acid precursors, GSLs are usually classified into aliphatic GSLs (methionine, alanine, leucine, isoleucine, and valine), indole GSLs (tryptophan), and benzenic GSLs (phenylalanine and tyrosine). The biological effects of GSLs are mainly attributed to their breakdown products, especially ITCs. GSL breakdown is facilitated by endogenous enzymes known as myrosinases (thioglucoside glucohydrolases (TGGs), EC 3.2.1.147) [3,4]. These enzymes are stored separately from GSLs but catalyze GSL hydrolysis when tissue damage occurs. In this TGG-dependent GSL hydrolysis, GSLs can yield ITCs, simple nitriles, epithionitriles, or thiocyanates, influenced not only by the GSL structure but also by the type of specifier protein (epithio-specifier protein (ESP), nitrile-specifier protein (NSP), and thiocyanate-forming protein), pH, and Fe^2+^.

The breakdown products of GSLs, particularly ITCs, have been extensively studied for their health benefits, including their anti-carcinogenic and anti-inflammatory properties, as well as their protection against cardiovascular diseases [1,5]. Sulforaphane, derived from glucoraphanin (GRA) according to TGG hydrolysis, is well known for its chemopreventive and anti-inflammatory properties, in vitro, in vivo, and in preclinical trials [1,5,6]. Erucin, another ITC hydrolyzed from glucoerucin (GER), also shows various health benefits [7,8]. Furthermore, in vivo studies have shown the inter-conversion between sulforaphane and erucin in human subjects [9,10,11]. However, simple nitriles and epithionitriles from GSLs have shown limited ability to induce quinone reductase or glutathione *S*-transferase or inhibit cancer cell growth [12,13]. Moreover, some simple nitriles may even have genotoxic effects in HepG2 cells [14]. Notably, some intact GSLs themselves have recently been found to have health-promoting effects. For example, GER possesses good direct as well as indirect antioxidant activity and effectively decomposes hydrogen peroxide and alkyl hydroperoxides [15]. Furthermore, GRA and GER could up-regulate cytochrome P450 and phase II enzyme systems in precision-cut rat lung slices [16]. However, the bioavailability of ITCs from fresh broccoli sprouts is dramatically higher than the broccoli supplements lacking TGGs [9]. Given the varying biological effects of GSL metabolites, we need to clarify the changing patterns of these health-promoting GSL profiles and their breakdown products during broccoli sprouting.

GRA and GER are aliphatic GSLs derived from methionine and transplanted from biosynthesis cells to seeds [17,18]. In plants, GER can be catalyzed by a subclade of flavin-monooxygenases into GRA [19]. GRA and GER are the predominant health-promoting GSL profiles and greatly accumulate in broccoli seeds [20,21]. However, compared to GRA and sulforaphane, GER and erucin in broccoli sprouts have received relatively little attention. Previous studies have reported genetic variation in GSLs and their breakdown products in broccoli seeds [20] and sprouts [22]. Some studies have highlighted the role of BolESPs in promoting the production of nitriles in broccoli florets [12,13] and sprouts [23], as well as Chinese kale sprouts [24]. However, some research on *Arabidopsis* has shown that some NSPs also play a role in nitrile production during GSL hydrolysis [25,26]. Nevertheless, there is limited knowledge regarding the variations in the GRA and GER breakdown products and the genes involved in TGG-dependent hydrolysis across the different developmental stages of broccoli sprouts. 

In a previous study, we selected some good varieties suitable for sprout production because their seeds contained high levels of GRA and GER but no alkenyl GSLs [20]. In this study, we utilized such one variety to investigate the variations in health-promoting GRA and GER in the sprouts, along with their breakdown products. Based on RNA-seq data, a comprehensive analysis of the gene expression related to GSL metabolism during broccoli sprout development was conducted.

## 2. Materials and Methods

### 2.1. Plant Materials and Cultivation 

The broccoli variety (1001) used in this study was a high-generation inbred line (>F_10_) with high levels of GRA and GER in its seeds, which also showed a high ratio of ITCs derived from GRA and GER [20]. The seeds were soaked in water for 2 h and then transferred onto non-woven fabric wetted with water in a clear plastic box. The box was closed and placed in a culture room at 25 °C with a 12 h light/12 h dark photoperiod. Water was added to the fabric to keep it moist. A total of 10 seedlings with no visible defects in their cotyledon shoot and root systems were randomly selected as normal seedlings and weighed for analysis. 

### 2.2. Chemicals and Reagents 

The *ortho*-nitrophenyl-D-galactopyranoside (*o*NPG), 2-propenyl ITC (≥99.7%), propyl-ITC (≥99%), and 4-pentenyl nitrile (≥97%) were purchased from Merck (Darmstadt, Germany). The 4-(methylsulfinyl)butyl ITC was purchased from Enzo Life Sciences GmbH (Lörrach, Germany). The 5-(methylsulphinyl)pentyl nitrile (sulforaphane nitrile) was chemically synthesized by Huafu Synthetic Chemicals (Hefei, China). Authentic GSLs were purchased from Phytoplan (Heidelberg, Germany). The methylene chloride (GC ultra grade), acetonitrile (ultra-gradient, HPLC-grade), and methanol (ultra-gradient, HPLC-grade) were purchased from Tedia Company (Fairfield, OH, USA). The water was of Milli-Q quality, and the individual GSLs were diluted with water and prepared as mother solutions (100 mM). All the GSL breakdown products were dissolved in methanol as mother solutions, which were diluted with dichloromethane (10 mM) for GC-FID analysis.

### 2.3. GSL Determination

GSL detection was performed according to our previous protocol with minor modifications [27]. Briefly, ten seedlings were weighed and moved into a 15 mL Eppendorf tube. Then, 5 mL boiling water was added, and the tube was immediately capped and immersed in boiling water for 15 min. After centrifuging at 12,000× *g* at 25 °C for 10 min, 2 mL of extraction solution was loaded into a polypropylene column (length, 6 cm, diameter, 0.5 cm) filled with diethylaminoethyl DEAE–Sephadex A-25 (Darmstadt, Germany), which had been activated with pyridine acetate (0.5 M), to a height of 1 cm. Then, the column was equilibrated twice with water (2 × 1 mL). The GSLs were converted into their desulfo analogs after 16 h of treatment or overnight with 100 μL 0.1% (1.4 U) sulfatase (Sigma-Aldrich, St. Louis, MI, USA) at room temperature. The desulfo GSLs were eluted with 2 × 0.5 mL water into 2 mL Eppendorf tubes.

The 1 mL elution was filtered through a 0.45 μm syringe filter for high-performance liquid chromatography (HPLC) analysis after the addition of *o*NPG as an internal standard. A Waters Breeze HPLC system (Waters, Milford, MA, USA) with an autosampler, a binary pump, and a UV–visible detector (models 717 Plus, 1525, 2487, respectively, Waters) was used for the GSL detection. A Spherisorb C18 column (5 µm, 250 × 4.6 mm i.d., Elite Analytical Instruments, Dalian, China) was used for separation at 25 °C. The mobile phases were water and acetonitrile. Separation was achieved with the following program: 5 min at 1.5% acetonitrile; 15 min gradient from 1.5% to 20% acetonitrile; 5 min at 20% acetonitrile; 1 min gradient to 100% acetonitrile; 5 min at 100% acetonitrile; 1 min gradient to 1.5% acetonitrile; and 8 min at 1.5% acetonitrile. The flow rate was 1.0 mL/min, and the injection volume was 20 μL. The concentrations of the GSLs were determined from the HPLC peak areas, the internal standards, and the published ultraviolet response factors of the individual desulfo GSLs at 226 nm [27]. 

### 2.4. GSL Breakdown Product Determination

The detection method for the breakdown products was performed according to our published method with some modifications [20]. Briefly, ten fresh seedlings were suspended in 1 mL of Millipore-filtered water and ground in a mortar. The slurry was transferred into a 15 mL capped tube, and the residue was washed with water (2 mL) twice. The combined solution was incubated at 25 °C for 2 h to facilitate GSL hydrolysis using endogenous TGG. After adding 0.2 µmol of the internal standard propyl ITC, dichloromethane (5 mL) was used for extraction using vortexing for 60 s and then centrifuging at 4000× *g* at 25 °C for 10 min. The extract (~1.8 mL) was centrifuged for 10 min at 12,000× *g*. The supernatant (~1 mL) was transferred into a 2 mL autosampler vial for GC-FID detection using an Agilent 7890A Series GC System (Agilent Technologies, Waldbronn, Germany) with an HP5 column (30 m, 0.25 mm, 0.25 m film), splitless injection at 200 °C, and the following temperature program: 50 °C for 3 min, a 20 °C/min ramp to 100 °C, a 3 °C/min ramp to 180 °C, hold for 10 min, an 8 °C/min to 220 °C, hold for 10 min, and then a 20 °C/min ramp to 230 °C (with a 1 min post-run at 240 °C). Hydrogen was used as the carrier gas with an injection volume of 1 μL and a flow rate of 1.5 mL/min. Quantification of the breakdown products was based on the standard curves of the authentic standards. An equal amount of sulforaphane was used with erucin, and an equal amount of sulforaphane nitrile was used with erucin nitrile. Six replicates were performed for each sample.

### 2.5. RNA-Seq Analysis

#### 2.5.1. RNA Extraction 

Total RNA was isolated from the sprouts (at 1 d, 3 d, and 5 d) using TRIzol^®^ Reagent according to the manufacturer’s instructions (Invitrogen, Waltham, MA, USA), and genomic DNA was eliminated using DNase I (Takara, Japan). Then, the RNA quality was determined using a 2100 Bioanalyzer (Agilent Technologies, Waldbronn, Germany) and quantified using the ND-2000 (NanoDrop Technologies, Pittsfield, MA, USA). A high-quality RNA sample (OD_260/280_ = 1.8~2.2, OD_260/230_ ≥ 2.0, RIN ≥ 6.5, 28S:18S ≥ 1.0, >10 μg) was used to construct the sequencing library. A total of 20 seedlings were taken together as one biological repeat. Three biological repeats were performed for each time point.

#### 2.5.2. Library Preparation 

Illumina HiSeq sequencing RNA-seq transcriptome libraries were generated using the TruSeq^TM^ RNA sample preparation kit from Illumina (San Diego, CA, USA) and 1μg of total RNA. In brief, mRNA was isolated with polyA selection using oligo(dT) beads and fragmented using fragmentation buffer. cDNA synthesis, end repair, base addition, and ligation of the Illumina-indexed adaptors were performed according to Illumina’s instructions. The libraries were then size-selected for cDNA target fragments of 200–300 bp on 2% Low Range Ultra Agarose, followed by PCR amplification using Phusion DNA polymerase (NEB) for 15 PCR cycles. After quantification using TBS-380, paired-end libraries were sequenced using the Illumina NovaSeq 6000 sequencing system (BIOZERON Co., Ltd., Shanghai, China). 

#### 2.5.3. Read Quality Control and Mapping

The raw paired-end reads were trimmed and quality-controlled using Trimmomatic with the parameters SLIDINGWINDOW:4:15, MINLEN:75 (version 0.36, http://www.usadellab.org/cms/index.php?page=trimmomatic, accessed on 30 June 2022). Then, clean reads were separately aligned with a reference genome (the HDEM *B. oleracea* reference genome, https://www.genoscope.cns.fr/projet_BKL/cgi-bin/gbrowse/boleracea/, accessed on 30 June 2022) in orientation mode using the HISAT2 (https://daehwankimlab.github.io/hisat2/, accessed on 30 June 2022) software. This software was used for mapping with the default parameters. Quality assessment of these data were undertaken using qualimap v2.2.1 (http://qualimap.bioinfo.cipf.es/, accessed on 30 June 2022). The gene reads were counted using HTSeq (https://htseq.readthedocs.io/en/release_0.11.1/, accessed on 30 June 2022). The sequences of the homologous ESP and NSP genes based on the HDEM reference genome were blasted in the Brassica Database (http://brassicadb.cn/#/, accessed on 30 June 2022).

#### 2.5.4. Differential Expression Analysis and Functional Enrichment 

To identify the DEGs (differential expression genes) between the two different samples (3 d vs. 1 d, 5 d vs. 1 d, 5 d vs. 3 d), the expression level for each gene was calculated using the fragments per kilobase of exon per million mapped reads (FRKM) method, which was performed by BIOZERON (Shanghai, China). The R statistical package edgeR (Empirical analysis of Digital Gene Expression in R, http://www.bioconductor.org/packages/release/bioc/html/edgeR.html/, accessed on 30 June 2022) was used for the differential expression analysis. In order to determine the DEGs, the following criteria were used: the logarithmic of fold change should be greater than 2, and the false discovery rate (FDR) should be less than 0.05. To understand the functions of the GEGs, GO functional enrichment and KEGG pathway analysis were carried out using goatools (https://github.com/tanghaibao/Goatools, accessed on 30 June 2022) and KOBAS (http://bioinfo.org/kobas/, accessed on 30 June 2022). DEGs were significantly enriched in GO terms and metabolic pathways when their Bonferroni-corrected *p*-value was less than 0.05.

### 2.6. RT-PCR Analysis

Total RNA was extracted using TRIzol Reagent according to the manufacturer’s instruction (Takara Technology, Dalian, China). PrimeScript RT Master Mix (Takara Technology, Dalian, China) was used to reverse-transcribe the mRNA into cDNA. qPCR was carried out using the CFX96 Real-Time PCR Detection System (Bio-Rad, Hercules, CA, USA). The primer sequences of the *BolNSP*s for RT-PCR were designed using the Primer-BLAST on NCBI (https://www.ncbi.nlm.nih.gov/, accessed on 30 June 2022) and are listed in Table S1. The primers for *BolESP*s were designed in a previous study [24]. The relative expression level of the target genes was computed using the 100 × 2^−ΔCT^ method according to our previous protocol [28]. 

### 2.7. Data Analysis 

Data analysis and graph creation were carried out using Microsoft Office Excel 2010. The heatmap of the gene expression was visualized using TBtools-II.

## 3. Results

### 3.1. Variation in GRA and GER

**Figure 1:**
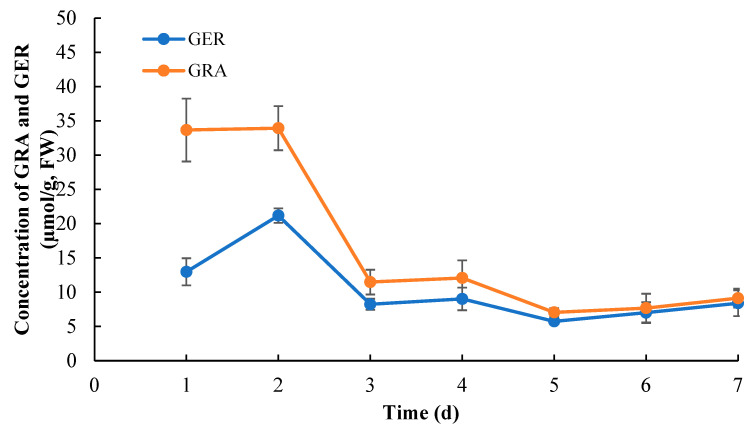
Changes in GRA and GER concentrations during the broccoli sprout development. GRA: glucoraphanin; GER: glucoerucin. Values are expressed as mean ± standard deviation (SD) (n = 6).

Our findings illustrated the concentrations of GRA decreased sharply from 33.66 µmol/g to 11.48 µmol/g and GER from 12.98 µmol/g to 8.23 µmol/g, respectively, after seed germination (1 d–3 d; Figure 1). Then, the concentration of GRA and GER, ranging from 12.07 µmol/g to 7.06 µmol/g for GRA and from 9.02 µmol/g to 5.75 µmol/g for GER, were maintained as relatively stable with a slight enhancement from 5 d to 7 d. Taken together, the concentrations of GRA and GER declined in the fresh broccoli sprouts as the seedlings aged.

### 3.2. Variation in GRA and GER Breakdown Products

**Figure 2:**
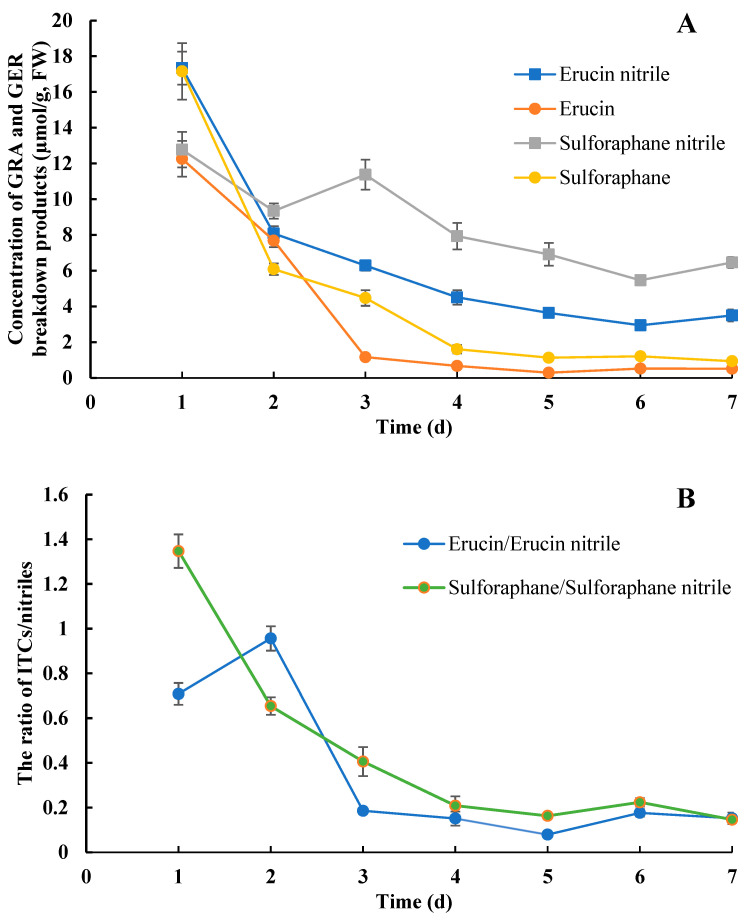
Changes in ITCs and nitriles derived from GER and GRA during broccoli sprout development. (**A**) The concentrations of erucin, erucin nitrile, sulforaphane, and sulforaphane nitrile; (**B**) the ratio of erucin/erucin nitrile and sulforaphane/sulforaphane nitrile. Values are expressed as mean ± SD (n = 6).

At the early stage of germination (1d), the levels of sulforaphane and erucin were 17.16 µmol/g and 12.26 µmol/g, respectively, basically equivalent to their corresponding nitriles (Figure 2A). However, the contents of sulforaphane ranged from 4.47 to 0.94 µmol/g in the broccoli sprouts after 3 d, lower than the corresponding sulforaphane nitrile levels (11.37 µmol/g to 5.46 µmol/g). A similar change pattern was closely followed by erucin and erucin nitrile, which ranged from 1.16 µmol/g to 0.29 µmol/g and from 6.29 µmol/g to 2.94 µmol/g, respectively. These results suggested that the major breakdown products of GRA and GER in the broccoli sprouts were nitriles rather than ITCs. 

On the other hand, all four breakdown products displayed a similar decline during broccoli sprouting. In order to elucidate the changes in the capacity for ITC production, we also calculated and analyzed the ratio of ITC/nitrile. The results showed that the ratio of sulforaphane to sulforaphane nitrile exhibited an obvious decline from 1 d to 5 d, keeping a low level from 3 d onward (Figure 2B). Erucin/erucin nitrile also exhibited a similar declining trend, except for from 1 d to 2 d. However, sulforaphane/sulforaphane nitrile were different from erucin/erucin nitrile during seedling development, especially during seed germination. 

### 3.3. Gene Expression in the GSL-TGG Pathway

**Figure 3:**
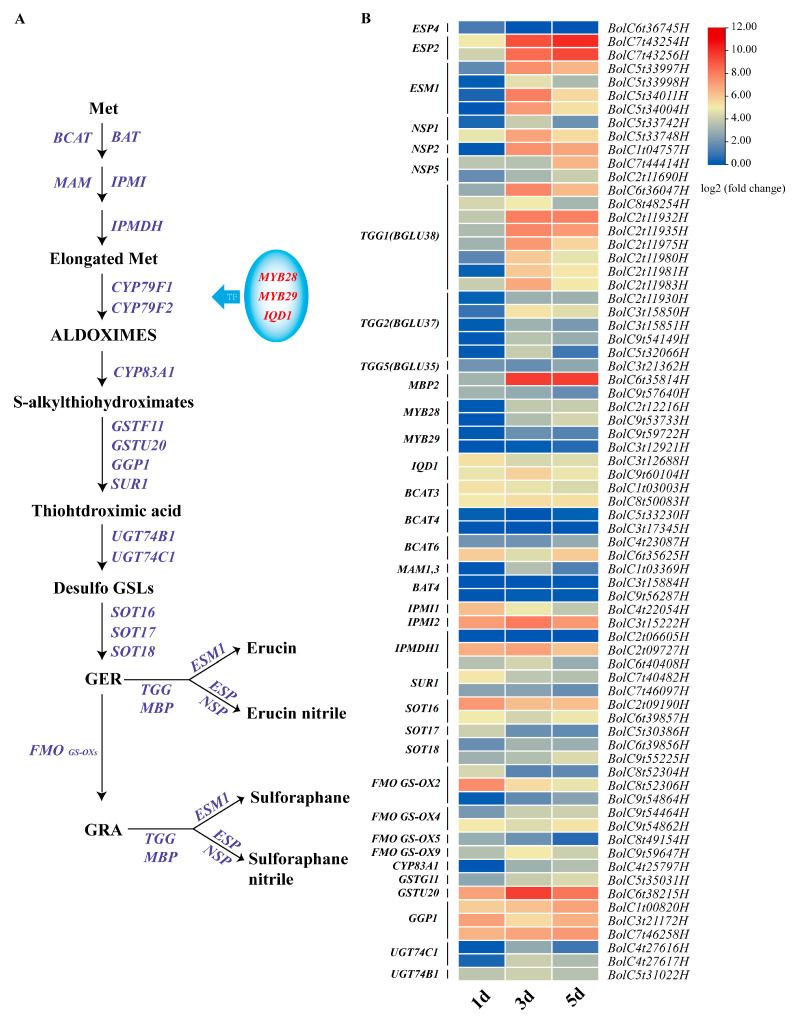
Transcriptomic analysis of TGG-dependent metabolic pathways of GRA and GER. (**A**) The GRA and GER metabolic pathways. (**B**) Analysis of gene expression in the GRA and GER metabolic pathways. Values represent the means of three replicates. The expression levels were visualized using TBtools. The heatmap scale ranges from −4 to +12 on a log2 fold change.

From 1 d to 5 d, the contents of GRA and GER and their breakdown products changed greatly. Thus, we conducted RNA-seq to reveal the changes in the GSL metabolism pathway in this period. The RNA-seq analysis showed 3519 up-regulated and 613 down-regulated genes in the 3d_vs_1d comparison, 333 up-regulated and 183 down-regulated genes in the 5d_vs_3d comparison, and 4070 up-regulated and 1778 down-regulated genes in the 5d_vs_1d comparison (Figure S1). The KEGG pathway enrichment analysis revealed that many genes were enriched in “metabolic pathways” and “biosynthesis of secondary metabolism” (Figure S2). In general, the DEGs in the 5d_vs_3d comparison were notably fewer compared to 3d_vs_1d and 5d_vs_1d, indicating a relatively stable development from 3 d to 5 d. The DEGs related to GSL metabolism were selected and analyzed (Table S2). A total of 43 genes (25 *Arabidopsis* homologous genes) related to GSL biosynthesis were identified. These genes, specifically involved in aliphatic GSL biosynthesis, such as *MYB28*, *CYP83A1*, and *MAMs* [1], had low expression levels at the beginning of seed germination but were slightly induced at 3 d and 5 d (Figure 3). Nevertheless, most genes, including *SUR1*, *SOT16*, *SOT18*, *GPP1*, *UGT74B1*, and *GST*s [1], involved in the core structure biosynthesis of both aliphatic GSL and indole GSL showed high expression from 1 d to 5 d (Figure 3). Similarly, transcription factors such as *MYB51*, *MYB122*, *MYB34*, *WRKY33*, and *MYC2*, as well as critical biosynthetic genes such as *CYP79B2* and *CYP83B1* involved in the indole GSL biosynthesis pathway [29], were up-regulated in broccoli sprouting (Table S2). 

**Figure 4:**
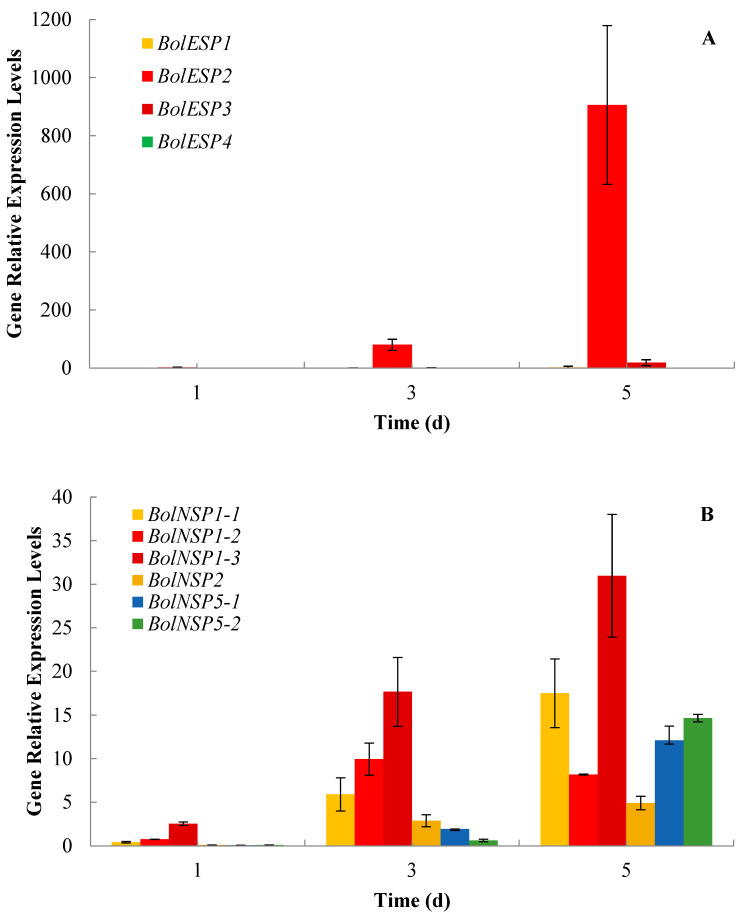
The relative expression levels of *BolESPs* (**A**) and *BolNSPs* (**B**) in broccoli sprouts. Values are expressed as mean ± SD (n = 3).

In comparison to biosynthesis and regulation, the genes involved in the hydrolysis of GRA and GER were up-regulated at the beginning of seed germination (Figure 3). Eight TGG1 homologous genes and five TGG2 homologous genes were identified. The current investigation revealed the high expression of TGGs at 3 d and 5 d, particularly *TGG1* homologous genes. Also, two homologous genes (*BolC4t28607H* and *BolC4t22177H*) of *BGLU28* were observed with a high expression from 1 d to 5 d. In addition, the RNA-seq analysis showed that two *ESP2* homologous genes (*BolC7t43254H* and *BolC7t43256H*) exhibited higher expression levels and gradually increased from 1 d to 5 d (Figure 3), which was confirmed using qRT-PCR (Figure 4A). Our results also showed that all four *EMS1* homologous genes were highly expressed at 3 d and 5 d. In this study, apart from one *BolNSP1* (*BolC5t33748H*) and two *BolNSP5* (*BolC7t44414H* and *BolC2t11690H*) genes expressed at 1 d, all the *BolNPS* genes identified in the samples using RNA-seq were highly expressed at 3 d and 5 d, which was verified using qRT-PCR (Figure 4B). 

## 4. Discussion

According to many previous studies, the anti-nutrient GSL profile progoitrin was widely detected in the seeds and sprouts of many broccoli cultivars [21,27,30]. In order to investigate the variations in GRA and GER as two major health-promoting GSL profiles in broccoli sprouts, the broccoli variety 1001 containing high levels of GRA and GER with progoitrin absent in its seeds [20] was used in this work. The results on the variation in GRA and GER in the broccoli sprouts were basically consistent with earlier studies [31,32,33]. The substantial decrease in the content of GRA and GER from 3 d to 6 d was observed in this study and a previous report [34]. Pérez-Balibrea et al. (2008) reported a similar trend in both light and dark conditions, with a general reduction in the concentration of aliphatic GSLs during a 7 d period [35]. Williams et al. (2008) found the GRA loss from 2 d to 7 d in broccoli sprouts (dry weight) was 38% for cv. Calabrese, 15% for cv. DeCicco, and 25% for cv. Romanesco [23]. In *Arabidopsis*, from 2 d to 8 d of seed-to-seedling development, the total GSL content per individual decreased by 30%, indicating GSL turnover [36]. Our previous study on Chinese kale sprouts also demonstrated significant variations in the total GSL content per plant as the sprouts developed [24]. All in all, the fresh broccoli sprouts (3 d–7 d) contained lower concentrations of GRA and GER compared to the germinating seeds (1 d), even though the older seedlings (5 d–7 d) had the potential to produce more GRA and GER than the early-aged seedlings (1 d–3 d). It has been reported recently that sulfur sources in aliphatic GSLs are reallocated by BGLU28 and BGLU30 under sulfur deficiency in the process of seed germination, particularly during early development in Arabidopsis [37]. Therefore, the main loss of GRA and GER may be a consequence of sulfur reallocation during the early stages of seed germination. 

We found that the concentrations of the breakdown products were much lower compared to the concentrations of GRA and GER at the same time point, and nitriles were the main breakdown products in broccoli seedlings. For consumers, it would be a better choice to intake sprouts treated with boiling water instead of fresh sprouts directly, when GRA and GER can be converted into ITCs by the gut microbes or there is extra intake of TGGs. Hansen and Schreiner (2017) also reported the contents of sulforaphane were no more than 1.2 µmol/g in 7–9-day-old seedlings from three varieties [22]. Moreover, they found the levels of sulforaphane nitrile and erucin nitrile were higher than the corresponding ITCs, especially in Marathon and Iron Man. Similarly, Williams et al. (2008) found the primary breakdown product of GRA was sulforaphane nitrile in broccoli seedlings of the Calabrese cultivar from 2 d to 7 d [23]. And the highest level of sulforaphane was lower than 1.5 µmol/g in fresh broccoli sprouts. They also observed a general decrease in the sulforaphane and sulforaphane nitrile concentrations during the first 7 days of seedling development based on fresh weight. However, Gu et al. (2012) noted that the content of sulforaphane declined sharply during the first day of seed germination based on dry weight and then increased to the same level as that in the seeds before decreasing again [38]. Wittstock et al. (2016) suggested that breakdown product analysis was best conducted with fresh plant material, as specifier-protein-mediated product formation was sensitive to freezing [4].

In addition, we found that sulforaphane/sulforaphane nitrile was different from erucin/erucin nitrile during seedling development, especially during seed germination. One possible reason is that the ESPs and NSPs have different binding affinity energies and affinity constants to GRA and GER [39]. In our study, the fresh sprouts were ground in water without adjusting the pH, nor adding other chemicals. According to previous studies, both a low pH (<5.0) and Fe^2+^ were able to assist TGG in producing nitriles with or without specifier proteins in vitro [40,41]. From a food nutrition perspective, it is recommended to harvest younger sprouts to provide more ITCs, which is also supported by our study on Chinese kale sprouts [24]. In the future, greater attention should be given to improving the ITC content and proportion using genetic and chemical methods.

The RNA-seq data revealed that the biosynthesis of aliphatic GSL was not activated significantly until 3 d. As is well known, chloroplasts are required for the side-chain elongation of aliphatic GSL in *Arabidopsis* [42]. Our further analysis also showed that many genes, such as *BolC4t27842H*, *BolC3t19882H*, and *BolC5t31588H*, related to the development of the chloroplasts were highly expressed at 3 d and 5 d but not at 1 d (Table S3). Therefore, it was not surprising that these aliphatic GSLs biosynthetic genes were activated after chloroplast formation, accompanied by a slight rise in GRA and GER in older broccoli sprouts. Different from aliphatic GSL, the biosynthesis of indole GSL was up-regulated after seed germination. These results verified a previous speculation that indole GSL accumulation in sprouts developing from 3 d to 11 d was possibly due to the activation of indole GSL biosynthesis [21].

Our earlier study demonstrated an increase in TGG activity from 3 d to 5 d in broccoli sprouts [33]. Correspondingly, we also found the TGG levels were high enough for GSL hydrolysis in broccoli seeds [20]. In this study, there was no GSL detected in the ground sprout slurry, indicating GSL had been degraded completely. Besides classical TGG, some atypical TGGs such as BGLU28 and BGLU30 [37] might also have the capacity to hydrolyze GRA and GER directly. In addition to TGGs themselves, some specifier proteins also play important roles in the TGG-GSL pathway by redirecting the breakdown products of GSL. For example, the functional ESP from the Arabidopsis ecotype Ler could convert alkyl GSLs into corresponding simple nitriles [40]. Furthermore, many studies have also highlighted the essential role of ESPs in promoting the production of nitriles from GRA in broccoli florets and sprouts [13,23,43]. Recently, we isolated four copies of *BolESPs* and found *BolESP2* was the most abundant isoform [24]. Silencing *BolESP2* not only effectively increased the total ITCs but also decreased the total epithionitriles in Chinese kale. So, we suspected that the two *BolESPs* might play important roles in promoting nitrile production in broccoli sprouts. However, it is well known that ESP activity can be suppressed by EPITHIOSPECIFIER MODIFIER1 (ESM1) [44]. This suggests that the effect of the *BolESP*s may have been partially suppressed by the highly expressed *BolESM1*s after seed germination, resulting in the production of ITC. Except for ESPs, prior research has confirmed the involvement of NSPs as cofactors in nitrile formation in *Arabidopsis*, notably NSP1, NSP2, and NSP5 [25,41,45]. The results of the up-regulation of several *BolNSPs* strongly suggested that these *BolNSP* genes likely promoted nitrile production during the hydrolysis of GSLs in broccoli sprouts. Similar to NSPs in protein structure, myrosinase-binding proteins (MBPs) also have jacalin domains. And they could form stable complexes with TGGs in *Brassica napus* [46,47]. A *BolMBP2* (*BolC6t35814H*) was highly expressed during broccoli sprout development, especially at 3 d and 5 d (Table S3). Its biological function remains unknown but is possibly associated with GSL hydrolysis. The RNA-seq data not only explained that the major breakdown products in broccoli sprouts were nitriles rather than ITCs but also illustrated the complexity of the TGG-GSL system in the development of broccoli sprouts.

## 5. Conclusions

Our results showed that the contents of GRA and GER, along with the proportion of ITCs, showed a constant decreasing trend during broccoli sprout development, and the main breakdown products from GRA and GER were nitriles instead of ITCs. Furthermore, transcriptome analysis revealed that specifier proteins, notably BolESP and BolNSP in the GSL-TGG pathway, likely contributed to the conversion of GSLs into nitriles in broccoli sprouts. In the future, proper pre-harvest and post-harvest handling and processing methods need to be explored to avoid or alleviate the loss of health-promoting GSLs in broccoli sprout production. More importantly, people should pay greater attention to promoting ITC formation during GSL hydrolysis.

## Data Availability

The authors confirm that the data supporting the findings of this study are available within the article and its Supplementary Materials.

## Supplementary Materials

The following supporting information can be downloaded at: https://www.mdpi.com/article/10.3390/plants13060750/s1, Figure S1: DEGs analysis for 3 d vs 1 d (up), 5 d vs 1 d (middle) and 5 d vs 3 d (down); Figure S2: KEGG analysis for 3 d vs 1 d (up), 5 d vs 1 d (middle), 5 d vs 3 d (down); Table S1: The primers designed and used for qRT-PCR in this study; Table S2: The data of transcriptome analysis. Table S3: The data of transcriptome analysis in TGG-GSL metabolic pathway.
