# Supplementary material for: Unravelling Glucoraphanin and Glucoerucin Metabolism across Broccoli Sprout Development: Insights from Metabolite and Transcriptome Analysis
Source: Plants (Basel). 2024 Mar 7;13(6):750. doi: 10.3390/plants13060750 (PMC10976094; doi:10.3390/plants13060750)
Supplement: Supplementary file 1 [file plants-13-00750-s001.zip › Figure S1 and S2.pdf]

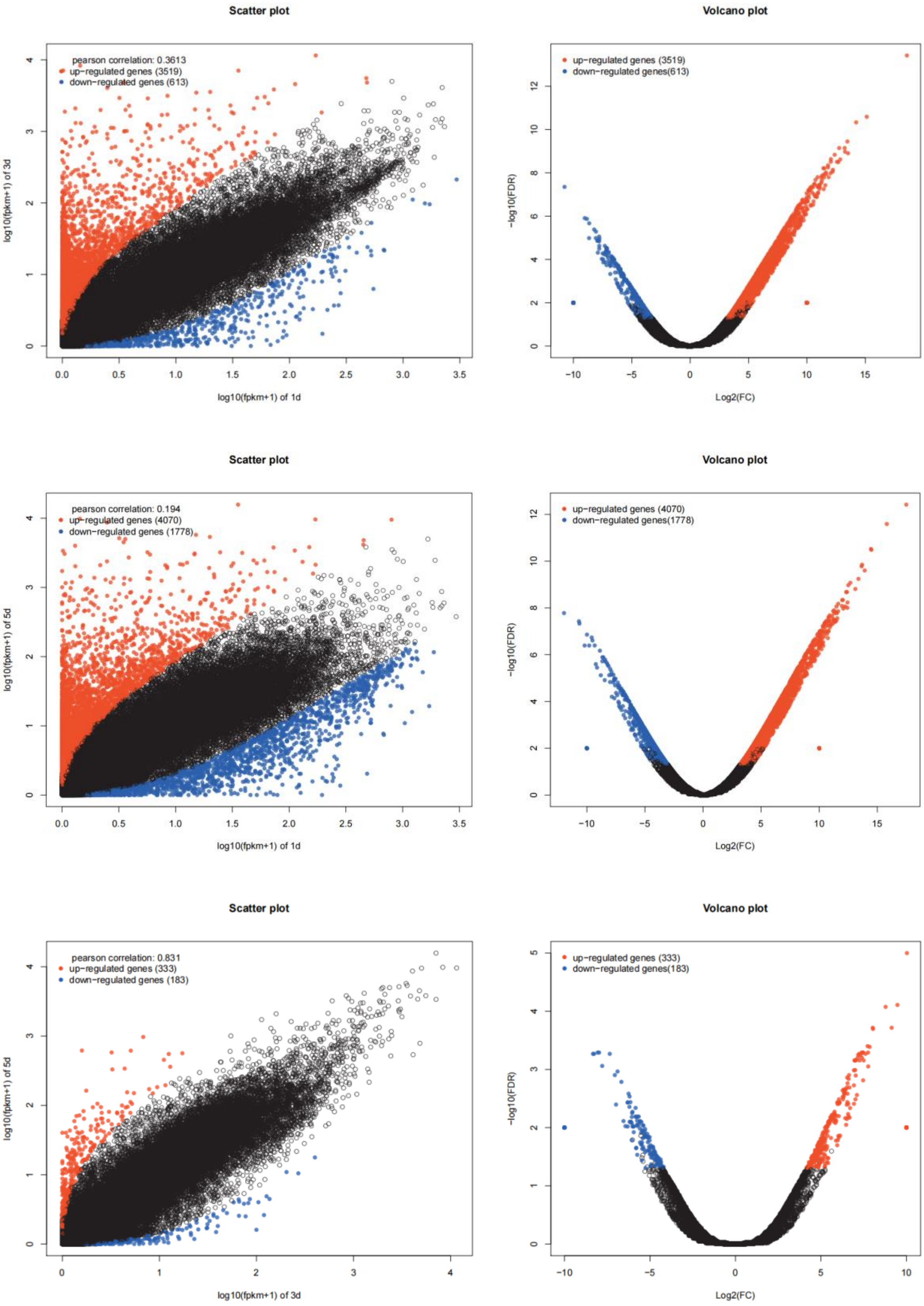

Figure S1. DEGs analysis for 3d vs 1d (up), 5d vs 1d (middle) and 5d vs 3d (down).

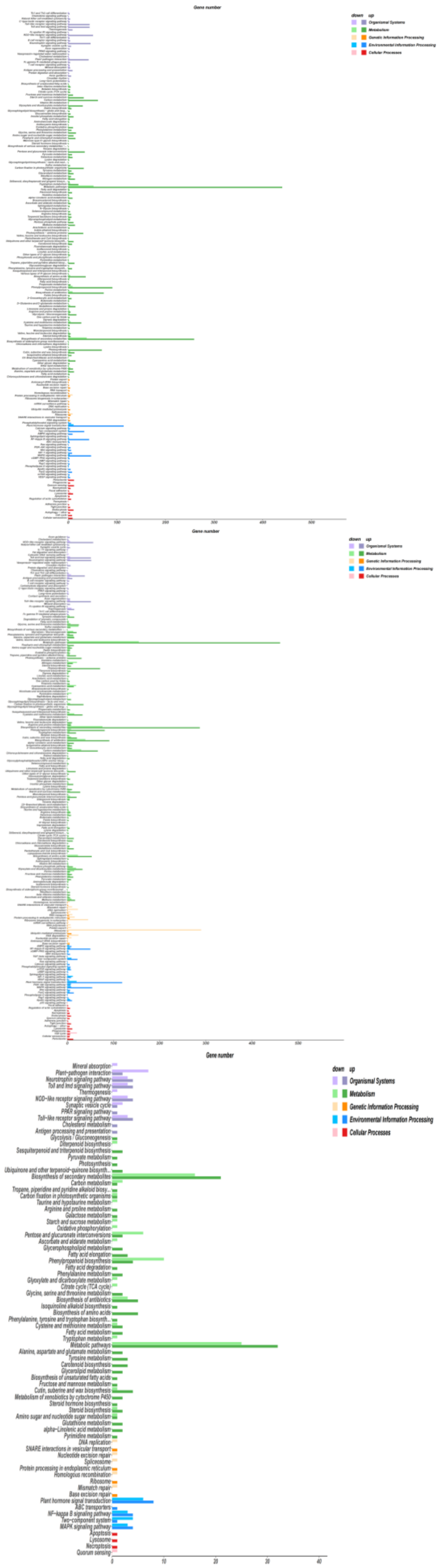

Figure S2. KEGG analysis for 3d vs 1d (up), 5d vs 1d (middle), 5d vs 3d (down).
