# Supplementary material for: Unravelling Glucoraphanin and Glucoerucin Metabolism across Broccoli Sprout Development: Insights from Metabolite and Transcriptome Analysis
Source: Plants (Basel). 2024 Mar 7;13(6):750. doi: 10.3390/plants13060750 (PMC10976094; doi:10.3390/plants13060750)
Supplement: Supplementary file 1 [file plants-13-00750-s001.zip › Table S1.pdf]

Table S1 The primers designed and used for qRT-PCR in this study.

Primers	Sequence(5'-3')	Length（bp）	Gene code	Gene name
757-F	CTGGGCTCGAAGTAGTGCAA	164	BolC1t04757H	*NSP2*
757-R	AACAACCGCACTAGCGAAGA			
742-F	GAAGTGGGTACAGTGTGCGA	90	BolC5t33742H	*NSP1-1*
742-R	ACCACCCAAACCTTCCCTTG			
748-F	AAACCCTTTTGGAGGCCACA	76	BolC5t33748H	*NSP1-2*
748-R	AGCTGTCCATCCCCTGATCT			
690-F	CGACGACACTCGCAGATACA	78	BolC2t11690H	*NSP5-2*
690-R	TCAACGGGAGACAGCAACTC			
753-F	TGGGTCCGGGACAATTGATG	76	BolC5t33753H	*NSP1-3*
753-R	TGTGGCCTCCAAAAGGGTTT			
414-F	CTGGGGAGGCTTGTAAAGGG	134	BolC7t44414H	*NSP5-1*
414-R	GGTCCATTTTCCGGTAGCCA			
